# Advances in research on medications for the treatment of alcohol use disorders: A review

**DOI:** 10.1097/MD.0000000000040045

**Published:** 2024-10-18

**Authors:** Yuzhou Chen, Luxin Zong, Qunde Zhao, Chuanxin Liu

**Affiliations:** a Jining Medical University, Jining, China; b Shandong Daizhuang Hospital, Jining, China.

**Keywords:** addiction, alcohol use disorder, mechanisms of action, pharmacotherapy

## Abstract

Alcohol use disorder (AUD) has become a global public health challenge, with the social and economic costs of alcohol abuse escalating, drawing widespread attention from healthcare professionals and the public worldwide. Particularly in recent years, the COVID-19 pandemic and associated lockdown measures have led to a significant increase in global alcohol consumption, further exacerbating the risk of AUD and sparking in-depth discussions on its prevention and treatment methods. Given that the current mainstay of AUD treatment relies on pharmacotherapy, the aim of this article is to review the progress of existing drug research for the treatment of AUD, and at the same time to focus on drug classes that have not yet been applied to the clinic, but have been shown in recent studies to be potentially exploitable for the treatment of AUD, in order to provide new ideas and directions for future drug research and development efforts.

## 1. Introduction

Alcohol use disorder (AUD) is widely recognized as a chronic, relapsing substance use disorder characterized primarily by the loss of control over alcohol use. This manifests as an intense craving for alcohol, uncontrollable drinking behavior, withdrawal symptoms upon reduction or cessation of drinking, and continued consumption despite severe negative consequences for one’s life. The complexity of this disorder lies in the pathological focus and dependence on alcohol that develops at the expense of other healthy behaviors, making it difficult for individuals to extricate themselves even upon recognizing the serious social, health, and economic consequences of their drinking.^[1]^

According to statistics from the World Health Organization, AUD is responsible for approximately 3 million deaths annually, accounting for 5.3% of total global mortality. This not only highlights the lethality of AUD but also reflects its broad socio-economic impact. Particularly among low-income and marginalized communities, the burden of AUD is especially heavy and closely linked to the average volume of alcohol consumed. In high-income and middle-income countries, the direct and indirect costs associated with alcohol, including health detriments, productivity losses, treatment expenses, and the social costs related to crime and violence,^[2]^ account for more than 1% of Gross Domestic Product.^[3]^

Moreover, the lockdown measures implemented during the COVID-19 pandemic have exacerbated the global issue of alcohol consumption, thereby increasing the risk of developing AUD. As people turned to alcohol more frequently as a coping mechanism for loneliness, anxiety, and uncertainty, alcohol consumption during the pandemic significantly rose, further intensifying the public health burden.^[4]^ A study using mortality analyses from the US National Center for Health Statistics demonstrated an increase in the number and rate of alcohol-related deaths of approximately 25% in 2019 to 2020 (the first year of the COVID-19 pandemic), and that the increase in alcohol-related mortality outpaced the increase in all-cause mortality by approximately 16.6% in 2020.^[5]^ Therefore, AUD is not only a threat to individual health but also a global public health issue, necessitating societal attention and comprehensive intervention measures.

## 2. Pharmacotherapy

Facing the global health challenge of alcohol dependency, finding and developing effective treatment methods has become a key task for the international medical community. Among various treatment strategies, pharmacotherapy has become a cornerstone due to its direct action on biological mechanisms, while the combined use of physical and psychotherapies provides diversified support for treatment. During the acute withdrawal phase, benzodiazepines such as diazepam and chlordiazepoxide are used to alleviate withdrawal reactions, supplemented by extensive administration of the B vitamins and symptomatic treatment for mental symptoms like impulsivity, laying the groundwork for treatment. In the post-acute phase, to prevent relapse into alcohol dependency, medications such as anti-craving drugs and alcohol-sensitizing drugs are introduced. These drugs reduce the desire to drink or increase discomfort upon alcohol consumption by modulating the activity of neurotransmitters or affecting the metabolic process of alcohol.

To date, the U.S. Food and Drug Administration (FDA) has only approved 3 drugs for treating alcohol dependency: acamprosate, disulfiram, and naltrexone, while the European Medicines Agency has also approved nalmefene.^[6]^ The approval of these drugs marks significant progress in treating AUD but also reflects the limitations of current pharmacological treatments. In recent years, the exploration of new drugs such as baclofen, topiramate, varenicline, and gabapentin has brought new hope to the field of AUD treatment.^[7]^ These potential therapeutic agents, acting through various mechanisms, could offer new treatment options for patients who do not respond well to existing therapies.

Given the limited efficacy of current treatment drugs, the research and development of new medications are particularly crucial. This article will review the latest research advances in currently available drugs, while focusing on drug classes that are not currently used in the clinic but have been shown in studies to be potential candidates for development, such as phosphodiesterase inhibitors and glucagon-like peptide-1 receptor agonists. The results and ideas from the latest research advances will provide new insights and further research directions for the pharmacological treatment of AUD, thus providing a more effective and convenient direction for future drug development.

### 2.1. Acamprosate

Acamprosate, as an advanced medication used in the treatment of AUD, demonstrates significant efficacy through its unique mechanism of action. The drug operates by antagonizing the N-methyl-D-aspartate receptors and indirectly affecting the neurotransmission of gamma-aminobutyric acid A, effectively alleviating the problem of glutamate system overactivation caused by alcohol intake. This regulation of the neurotransmitter system helps reduce the hyperactivated neurological state induced by alcohol,^[8]^ thus providing an effective treatment option for patients with AUD.

The most common side effects associated with acamprosate involve the nervous and digestive systems, including symptoms like dizziness and diarrhea.^[9]^ Despite these side effects, acamprosate is generally considered well-tolerated in clinical practice. Notably, a meta-analysis of 17 clinical trials highlighted acamprosate’s significant advantage over placebo in promoting sustained abstinence,^[10]^ further confirming its efficacy in reducing the risk of relapse post-detoxification for alcohol-dependent individuals.

Acamprosate attracts attention not only for its potential therapeutic effects on AUD but also for its good tolerance and significant efficacy demonstrated in clinical trials, making it an important treatment option. Nonetheless, monitoring its side effects remains an essential consideration in the treatment process, necessitating meticulous patient monitoring and management by medical professionals when using acamprosate to treat AUD.

### 2.2. Disulfiram

Disulfiram, an alcohol-sensitizing drug, operates on the principle of affecting the alcohol metabolism process. By inhibiting the activity of aldehyde dehydrogenase in the liver, disulfiram blocks the normal metabolic pathway from ethanol to acetic acid, leading to the accumulation of acetaldehyde in the body. This buildup of acetaldehyde triggers a series of uncomfortable reactions, such as tachycardia, facial flushing, nausea, and vomiting, typically occurring about 10 minutes after alcohol intake.^[11]^ This physical aversion effectively compels patients to avoid alcohol consumption, thus achieving the goal of abstinence. However, disulfiram faces challenges of limited effectiveness in actual clinical application.^[12]^

Clinical research and experimental results indicate that although disulfiram can induce discomfort following alcohol intake, its effect on inhibiting relapse behavior in patients is inconsistent. The results of a double-blind, randomized controlled trial showed that patients using disulfiram did not exhibit a significant deterrent effect on relapse compared to the control group, and the adverse reactions significantly increased among these patients.^[13]^ These findings highlight the limitations of disulfiram in treating alcohol dependency, particularly the uncertainty of its clinical efficacy and the risks of potential adverse reactions.

Although the mechanism of disulfiram may theoretically be effective for some patients, due to its limited efficacy and potential adverse reactions, this medication is no longer recommended as the first-line treatment method for most patients in the current alcohol dependency treatment practices.

### 2.3. Naltrexone

Since its approval by the FDA in 1994 for the treatment of AUD, naltrexone has become a crucial component of AUD treatment regimens. Its mechanism of action is believed to involve the antagonism of μ-opioid receptors, thereby reducing patients’ craving for alcohol, helping to maintain sobriety, and decreasing the amount of alcohol consumed. Numerous clinical trials have confirmed that naltrexone is more effective than placebo in helping patients reduce the frequency and quantity of drinking, demonstrating good therapeutic effects.^[14]^

Despite naltrexone’s significant efficacy in treating AUD, its use may be accompanied by some adverse reactions, the most common of which include gastrointestinal responses (such as nausea) and neurological reactions (such as dizziness). These uncomfortable symptoms somewhat limit naltrexone’s application range and patient tolerance.

Nalmefene, as a newer opioid receptor antagonist with stronger affinity, longer duration of action, more administration routes, and lower risk of adverse reactions, is somewhat considered an upgraded alternative to naltrexone. The advantages of nalmefene make its application in treating AUD more widespread, particularly offering another effective treatment option for those who experience adverse reactions to naltrexone or have poor treatment outcomes.

In summary, naltrexone, as a medication for treating alcohol dependence, has proven its effectiveness in reducing cravings for alcohol and the amount of alcohol consumed by patients.

### 2.4. Topiramate

Topiramate, primarily utilized in clinical settings for seizure treatment,^[15]^ has garnered interest from the scientific community for its potential in treating AUD due to its neuropharmacological mechanisms similar to those of alcohol. Topiramate can reduce brain excitability by inhibiting glutamatergic receptors and enhancing γ-aminobutyric acid neurotransmission,^[16]^ paralleling the neuroinhibitory effects of alcohol. Unlike alcohol, however, topiramate does not lead to issues of abuse or dependency, making it a potentially safe option for treating AUD.

Although not yet approved by the FDA for AUD treatment, existing studies show positive effects of topiramate on AUD. Particularly, results from double-blind, randomized controlled trials indicate significant improvements in patients treated with topiramate, including reduced average alcohol consumption, fewer drinking days, and an increase in abstinent days.^[17]^ These findings provide preliminary scientific evidence for the use of topiramate in AUD treatment and pave the way for future therapeutic research.

Despite the potential therapeutic effects shown in early studies, the widespread clinical application of topiramate for AUD treatment faces challenges. More extensive and long-term clinical trials are needed to verify its safety and efficacy. Furthermore, the action mechanism, optimal dosage, and treatment protocols of topiramate require further clarification. Finally, considering topiramate’s primary use and its impact on the neurological system, a detailed assessment of potential side effects and impacts of long-term use is necessary.

Topiramate presents as a promising medication for AUD treatment, warranting further exploration and verification through additional research. Future studies are expected to provide more treatment options and hope for alcohol dependency, offering more effective therapeutic methods for patients.

### 2.5. Baclofen

Baclofen, an agonist at GABAB receptors, has recently been the focus of AUD treatment research due to its potential mechanism of treating AUD through the activation of GABAB receptors in the midbrain limbic circuits and the ventral tegmental area, subsequently inhibiting dopamine release.^[18]^ This process plays a crucial role in reducing alcohol-induced reward and craving sensations.

In clinical trials, baclofen has shown potential, with randomized controlled trials indicating its effectiveness in helping patients reduce alcohol consumption and alleviate cravings and anxiety symptoms associated with AUD. However, studies also reveal inconsistencies in baclofen’s effectiveness on AUD patients, highlighting the complexity and uncertainty regarding its therapeutic impact on AUD.

Currently, there is insufficient and inconsistent evidence supporting the efficacy and safety of baclofen in treating AUD, mainly due to small sample sizes, variations in study design, and significant outcome differences among existing studies. Therefore, more large-scale, high-quality clinical controlled research is needed to explore and validate baclofen’s role and effectiveness in AUD treatment comprehensively.

In summary, while baclofen as a potential medication for treating AUD shows promise, it also faces significant challenges. Future research needs to address these inconsistencies through more rigorous and comprehensive study designs to understand baclofen’s therapeutic mechanisms, indications, dose–response relationships, and long-term safety, thereby providing more effective and safer treatment options for AUD patients.

### 2.6. Varenicline

Primarily used as an aid for smoking cessation, varenicline has drawn attention for its ability to reduce the rewarding effects of smoking by activating specific nicotinic acetylcholine receptors. Its mechanism involves binding to α4β2 nicotinic acetylcholine receptors, slowing the dopamine release triggered by receptor activation, similar to the process induced by alcohol.^[19]^ Based on this similarity, researchers have begun exploring varenicline’s potential utility in treating AUD.

Recent studies show that although varenicline did not significantly differ from the placebo in maintaining abstinent days, it effectively reduced patients’ average daily alcohol consumption.^[20]^ Especially in a double-blind controlled trial, patients treated with varenicline experienced significant reductions in heavy drinking days per week, daily drinking quantity, and craving for alcohol compared to those receiving a placebo,^[21]^ indicating varenicline’s potential in reducing drinking behavior in AUD patients.

Despite some positive preliminary results in treating AUD, varenicline’s efficacy and safety as an AUD treatment require further validation through more extensive, larger-scale clinical controlled studies. Specifically, detailed examination of varenicline’s therapeutic effects, optimal dosage, treatment duration, and long-term safety are crucial.

Varenicline, as an emerging potential option for AUD treatment, holds promise in reducing alcohol consumption and controlling cravings. Future research could provide a firmer scientific basis for varenicline’s therapeutic application and potentially offer more treatment choices for AUD patients, thereby improving their treatment outcomes and quality of life.

### 2.7. Gabapentin

Gabapentin, an oral medication primarily used for treating seizures, has recently drawn attention for its potential use in treating AUD. Despite the exact mechanism of action not being fully understood, meta-analyses have shown gabapentin to have a moderate effect on reducing the number of heavy drinking days compared to a placebo. However, for other treatment outcomes like achieving complete abstinence, recurrence of heavy drinking, days without drinking, daily alcohol consumption, and improvements in gamma-glutamyl transferase (a marker for liver function) concentrations, gabapentin did not show significant effects.^[22]^

Additionally, some adverse events have been reported with gabapentin use, especially at higher doses, including neurological symptoms like dizziness and somnolence, as well as peripheral edema.^[23]^ These side effects limit gabapentin’s potential application in treating AUD, particularly when weighing the benefits against potential risks.

Current research on gabapentin’s efficacy and safety in treating AUD is still relatively limited, necessitating further clinical trials and studies. This includes determining optimal dosages, treatment durations, and the safety of long-term use to provide more effective and safer treatment options for AUD patients.

Gabapentin shows promise as a potential AUD treatment, particularly in reducing heavy drinking days, but its impact on other treatment outcomes is not significant, and there are risks of side effects. Future research should delve deeper into the value of gabapentin in AUD treatment and how to optimize its treatment protocol to maximize efficacy while minimizing potential side effects.

### 2.8. Glucagon-like peptide-1 (GLP-1) receptor agonists

GLP-1 receptor agonists, primarily utilized in the management of type 2 diabetes and obesity due to their significant effects on appetite control and weight management, especially semaglutide, a GLP-1 analog, have recently garnered widespread attention. Beyond their primary clinical applications, the potential utility of GLP-1 receptor agonists in substance dependency, particularly in the treatment of alcohol dependency, has begun to be explored by the scientific community.

Early laboratory studies, including those conducted on mice, rats, and non-primate animals using various alcohol-related models, have evaluated the impact of GLP-1 receptor agonists. These studies have discovered that GLP-1 receptor agonists can diminish the rewarding properties of alcohol and other addictive substances, such as nicotine, opioids, and cocaine, thereby reducing intake and dependency motivation. These findings provide preliminary experimental support for the potential of GLP-1 receptor agonists in treating substance dependency, notably alcohol dependency.

Recent evidence-based medical research further indicates that patients seeking treatment with semaglutide exhibit reduced cravings and desires for alcohol. These results suggest that semaglutide and other GLP-1 receptor agonists could represent a new approach to treating alcohol dependency. However, despite these positive early studies and clinical observations, significant challenges must be overcome before GLP-1 receptor agonists can be broadly applied in alcohol dependency treatment. Specifically, there is a lack of large-scale clinical controlled studies on addiction patients, and the safety and efficacy of these drugs in substance dependency treatment have not been fully verified.^[24,25]^

Therefore, future research needs to focus on collecting more data on the effectiveness and safety of GLP-1 receptor agonists in treating alcohol dependency through clinical trials. This includes evaluating their impact on reducing alcohol intake, controlling alcohol cravings, improving alcohol dependency-related behavioral and physiological indicators, and monitoring potential adverse reactions. Through these studies, the scientific community can gain a more comprehensive understanding of the potential of GLP-1 receptor agonists in treating alcohol dependency, offering more treatment options for patients.

### 2.9. Ketamine

The approval of esketamine, a ketamine enantiomer, for the treatment of treatment-resistant depression marks significant recognition of the therapeutic potential of this medication. Depression is a common comorbidity among individuals with AUD, often making it more difficult for these patients to abstain due to depressive symptoms, and the presence of depression itself may increase the risk of alcohol relapse. In this context, the application of ketamine and its enantiomer, esketamine, offers a potential treatment pathway not only for treatment-resistant depression but also brings new hope for patients with AUD.

Ketamine’s mechanism of aiding abstinence through the alleviation of depressive symptoms,^[26]^ coupled with its potential for promoting neural regeneration, provides a unique treatment opportunity for AUD. Preclinical evidence suggests that ketamine can facilitate the regeneration of synapses and neurons damaged by addiction,^[27]^ which could significantly improve treatment outcomes for AUD patients.^[28]^

A double-blind controlled clinical trial investigating ketamine’s efficacy in treating AUD further explored this potential. The study found that patients receiving ketamine-assisted psychotherapy showed more abstinent days over 3 and 6 months of follow-up compared to those receiving placebo psychotherapy, though the overall relapse rates between the 2 groups did not significantly differ. These results suggest that ketamine holds promise in aiding AUD treatment, but also emphasize the need for more extensive clinical trials to confirm this.^[29]^

The promise of ketamine for the treatment of AUD is supported by studies with more significant treatment effects, especially when compared to existing treatments. Studies have shown ketamine to be effective in prolonging abstinence in abstinent alcoholics, for example, Krupitsky and Grinenko (1997) study found that treatment with ketamine increased one-year abstinence rates in alcoholics from 24% in the control group to 66% in the ketamine group, and reduced abstainers’ cravings for alcohol, which in turn lowered the likelihood of relapse. Krupitsky and Grinenko study also found that patients reported a deeper self-understanding of the negative aspects of alcohol dependence after ketamine treatment, leading to an increased commitment to abstinence. Among other things, ketamine works through a variety of mechanisms, including enhanced neuroplasticity and neurogenesis, rapid antidepressant effects, and blocking alcohol-related memory reconsolidation. These findings all clearly demonstrate that ketamine is orders of magnitude more effective than existing pharmacological treatments by a factor of 2 or even 3 in its therapeutic impact on AUD.^[30]^

Ketamine treatment was well-tolerated in subjects, with no serious adverse events related to the study drug and most ketamine-related adverse events assessed as mild compared to the placebo group. In addition, subjects in the ketamine group showed a more linear improvement in liver function indices during the study period compared to a U-shaped response in the placebo group, suggesting that ketamine may have a protective effect on liver function.^[29]^

While ketamine has shown initial positive results in terms of its promise for the treatment of AUD and its safety, more extensive research is needed to fully understand its potential in this area. Future research should aim to explore these medications’ application in treating AUD and related depressive symptoms more deeply, to provide more effective treatment options for patients.

### 2.10. Phosphodiesterase inhibitors

Advancements in the study of phosphodiesterase-4 (PDE4) inhibitors have indicated a potential breakthrough in the treatment of AUD. PDE4 is a specific cAMP hydrolase. By inhibiting PDE4, intracellular cAMP levels can be increased, and the increased cAMP activates protein kinase A, which in turn promotes phosphorylation of cAMP response element binding protein. Phosphorylated cAMP response element binding protein regulates the expression of a series of genes that are associated with neuroadaptive changes, such as neuropeptide Y, corticotropin-releasing factor, and brain-derived neurotrophic factor. These genes play an important role in the regulation of central nervous system function and alcohol-dependent behavior. Studies have shown that cAMP signaling is closely related to the positive and negative reinforcement mechanisms of alcohol drinking behavior. By increasing cAMP levels, PDE4 inhibitors can achieve a reduction in the positive reinforcement effects of alcohol, thereby reducing alcohol use and preference.^[31]^

Although most PDE4 inhibitors have been limited in their application due to significant clinical side effects, such as nausea and diarrhea, recent research has identified a new PDE4 inhibitor, compound 4-(6-chloro-4-ethoxy-1H-pyrazolo[4,3-c]pyridin-1-yl)butanoic acid (ZL 40), which exhibits favorable pharmacological characteristics and a lower risk of side effects. Zheng et al performed a series of chemical modifications to the indole backbone. By optimizing the substituent at the 4-position to ethoxy, the interaction with the hydrophobic pocket of the PDE4 enzyme active site was enhanced, while its smaller size and appropriate length allowed it to be properly embedded in the enzyme binding pocket, improving the overall stability and binding strength of the compound. By introducing the chlorine atom at the 6-position, the high electronegativity and smaller size of the chlorine atom help to optimize the electron distribution and steric structure of the molecule, which enhances the binding efficiency with PDE4. In addition, the chlorine atom at the 6-position may be involved in hydrogen bonding or van der Waals force interactions, which are essential for maintaining the positioning of the molecule in the PDE4 active site. It was shown that the side chain of the butyrate group at position N1 is believed to be able to form stable hydrogen bonds with the metal ion binding site of PDE4, thus significantly enhancing the inhibitory activity. Meanwhile, in order to further optimize the backbone structure, optimized alkyl carboxylic acid and hydroxamic acid derivatives were introduced at the N1 position, which are structurally able to effectively fill the binding pocket of PDE4, improve the binding and inhibition efficiency of the molecule, and provide a stronger interaction force compared to the other substituents, resulting in an improved inhibition activity. ZL40 was selected as the compound with the most significant PDE4 inhibitory activity and selectivity, which possessed highly potent PDE4 inhibitory activity.

And the newly discovered compound ZL40 is highly selective compared to other PDE4 inhibitors, ZL40 is a selective PDE4 inhibitor with significantly higher inhibitory activity (IC50 = 37.4 nM) against PDE4D than other isoforms. This selectivity helps to reduce interference with other PDE isoforms, thereby reducing the occurrence of side effects; secondly ZL40 demonstrated a good oral bioavailability (F = 94%), ensuring that ZL40 enters the blood system quickly and efficiently after administration and is effectively absorbed in the body. Its pharmacokinetics showed that in SD rats, ZL40 was detected at plasma, liver and brain concentrations at 1 or 2 hour time points, and despite lower concentrations in the brain, was able to reach a certain concentration level within 1 to 2 hours, with increasing concentrations over time. This efficient distribution property is essential for its use as an AUD therapeutic candidate. In addition, the long half-life (9.3 hours) and high plasma exposure of ZL40 further enhance its pharmacokinetic benefits in vivo, contributing to the drug’s efficacy and reducing the risk of side effects. Meanwhile ZL40 showed a satisfactory safety profile in vivo. It showed a significant reduction in alcohol intake and preference in animal models and improved alcohol-induced acute sedation and dyskinesia. The emetic potential was significantly reduced compared to approved PDE4 inhibitors such as roflumilast and apremilast. This suggests that ZL40 has the potential to treat AUD with no significant toxicity or adverse effects in the dose range. In conclusion, ZL40, as a novel PDE4 inhibitor, has high selectivity, good bioavailability, and satisfactory safety profile for the treatment of AUD compared with other PDE4 inhibitors, and these features help to reduce the risk of side effects and improve therapeutic efficacy.

Notably, its potential therapeutic effects in reducing alcohol intake, alleviating withdrawal anxiety behaviors, and attenuating alcohol-induced liver damage were demonstrated in multiple animal models. ZL40 reduced alcohol intake and preference in mice in time-restricted free choice and twice-in-a-row tests. Alcohol intake was significantly reduced after 2 and 3 weeks of treatment in the tests. The behavior of the animals demonstrated that ZL40 was effective in ameliorating symptoms of alcohol-induced dyskinesia and somnolence. During alcohol withdrawal, ZL40-treated animals showed shorter rest periods and longer distances in the open test arena, suggesting an ameliorative effect on anxiety-like behaviors. In the National Institute on Alcohol Abuse and Alcoholism mouse model, ZL40 significantly reduced liver indices (liver weight/body weight ratio) due to chronic alcohol feeding; lowered serum levels of alanine aminotransferase and aspartate aminotransferase (indices of liver injury); and, in addition, histopathological tests showed that ZL40 ameliorated liver injury and steatosis; Reduced serum levels of pro-inflammatory factors IL-6, TNF-α, and IL-1β, suggesting an anti-inflammatory effect; these findings support the potential application of ZL40 in the treatment of AUD, as it not only reduces the triggers for drinking, but also helps to alleviate the psychological and physiological symptoms associated with alcohol withdrawal, as well as protects the liver from alcohol damage.

This research is crucial for the development of new, effective, and lower side effect risk medications for AUD treatment. ZL 40, as a novel and effective PDE4 inhibitor, its excellent drug-like properties, and potential in treating AUD, heralds a hopeful future direction in addressing alcohol dependency and related diseases.^[32]^ Despite these encouraging findings, further clinical research is necessary to validate ZL 40’s safety, efficacy, and the impact of its long-term use in humans, aiming to transition this innovative treatment from the laboratory to clinical application successfully.

## 3. Summary and outlook

In the field of AUD treatment, recent research has made significant progress, uncovering various potential pharmacological mechanisms. These mechanisms primarily function through activating or inhibiting specific operable signaling pathways. GLP-1 agonists and PDE4 inhibitors have different mechanisms of action in the treatment of AUD. GLP-1 agonists reduce alcohol intake and its rewarding effects by slowing gastric emptying and increasing satiety, primarily through modulation of the ventral pallidum and the nucleus ambiguus within the central nervous system. Animal studies and preliminary human studies have shown that GLP-1 receptor agonists are effective in reducing alcohol intake, but more randomized controlled trials are needed to confirm their efficacy. On the other hand, PDE4 inhibitors increase intracellular cAMP levels, reduce inflammation and oxidative stress, and improve withdrawal symptoms and anxiety by inhibiting PDE4. Although animal studies have shown that PDE4 inhibitors are effective in reducing alcohol intake and ameliorating alcohol-induced negative effects, their clinical application is limited by their high emetogenicity, and further studies are needed to determine their safety and efficacy. In summary, GLP-1 receptor agonists and PDE4 inhibitors have their own advantages and disadvantages in modulating signaling pathways associated with the development of AUD, and future studies should further explore their potential for clinical application.

The development of new medicines for the treatment of AUD faces many challenges, including the lack of large-scale clinical trials, the complexity of evaluating the safety of the medicines, and the complexity of individualizing treatment. In the face of these challenges, however, there are also opportunities in this area such as diverse treatment pathways and individualized medicine due to technological advances. For example, identifying drug targets through known signaling pathways is not only an effective and rapid method but also helps shorten the time from drug discovery to clinical application, while improving the specificity and efficacy of the treatment. Furthermore, extensive clinical trials and comprehensive drug safety assessments are key to ensuring the effectiveness and safety of new medicines. Similarly, the importance of individualized treatment lies in the ability to tailor the most effective treatment regimen to different patients’ backgrounds and health conditions. Ways to improve patient prognosis include multidisciplinary collaborative learning studies, patient education and support, and continued close clinical monitoring and adjustment of treatment regimens.

Future research priorities may include: delving into the pathophysiological mechanisms of AUD, identifying new drug targets; conducting large-scale, multicenter clinical trials to validate the efficacy and safety of potential treatment drugs; and researching individualized treatment strategies, considering factors such as the patient’s genetic background, comorbidities, and lifestyle habits, to achieve more precise AUD treatment.

In conclusion, despite the challenges, the future of treating AUD looks promising with the advancement of science and technology and the innovation of research methods. Through interdisciplinary cooperation and international collaboration, pushing forward the discovery and development of new drugs is expected to bring more effective and safer treatment options for AUD patients.

## Author contributions

**Resources:** Luxin Zong, Qunde Zhao.

**Writing – original draft:** Yuzhou Chen.

**Writing – review & editing:** Yuzhou Chen, Chuanxin Liu.
